# Transcriptomic Response of Porcine PBMCs to Vaccination with Tetanus Toxoid as a Model Antigen

**DOI:** 10.1371/journal.pone.0058306

**Published:** 2013-03-25

**Authors:** Marcel Adler, Eduard Murani, Ronald Brunner, Siriluck Ponsuksili, Klaus Wimmers

**Affiliations:** 1 Leibniz Institute for Farm Animal Biology (FBN), Institute for Genome Biology, Dummerstorf, Germany; 2 Leibniz Institute for Farm Animal Biology (FBN), Research Group Functional Genome Analysis, Dummerstorf, Germany; Kyushu Institute of Technology, Japan

## Abstract

The aim of the present study was to characterize *in vivo* genome-wide transcriptional responses to immune stimulation in order to get insight into the resulting changes of allocation of resources. Vaccination with tetanus toxoid was used as a model for a mixed Th1 and Th2 immune response in pig. Expression profiles of PBMCs (peripheral blood mononuclear cells) before and at 12 time points over a period of four weeks after initial and booster vaccination at day 14 were studied by use of Affymetrix GeneChip microarrays and Ingenuity Pathway Analysis (IPA). The transcriptome data in total comprised more than 5000 genes with different transcript abundances (DE-genes). Within the single time stages the numbers of DE-genes were between several hundred and more than 1000. Ingenuity Pathway Analysis mainly revealed canonical pathways of cellular immune response and cytokine signaling as well as a broad range of processes in cellular and organismal growth, proliferation and development, cell signaling, biosynthesis and metabolism. Significant changes in the expression profiles of PBMCs already occurred very early after immune stimulation. At two hours after the first vaccination 679 DE-genes corresponding to 110 canonical pathways of cytokine signaling, cellular immune response and other multiple cellular functions were found. Immune competence and global disease resistance are heritable but difficult to measure and to address by breeding. Besides QTL mapping of immune traits gene expression profiling facilitates the detection of functional gene networks and thus functional candidate genes.

## Introduction

In pig farming, the incidence and severeness of infectious diseases has direct influence on animal welfare, product quality and economics. Since both intensive and organic production systems are faced with multiple infectious diseases, there is a need for animals endogenously protected against a broad range of pathogens.

Sustaining the pig’s genetic resistance to infection has been considered as a key breeding goal to improve disease prophylaxis [1]. Wilkie and Mallard [2] proposed indirect selection for general disease resistance to avoid that selection for resistance to specific infections or diseases lead to susceptibility to others.

Little is known about the genetic control within the relationship between immune traits and performance traits. In poultry where several studies are available the selection for high growth rates resulted adversely in an impaired immune competence [3], [4]. Vice versa, high immune responsiveness may be associated with a corresponding allocation of resources on the costs of productivity. However, in the pig selection for high immune response was associated with enhanced weight gain [5], [6]. Taken together, in order to identify genotypes for selection the investigation of underlying immunogenetic fundamentals plays a major role.

First studies of gene regulation during the porcine immune response focussed on peripheral blood mononuclear cells (PBMCs) so far were limited to candidate genes [7]–[10]. For the study of gene expression due to immune stimulation or pathogen infection microarray techniques enable the interrogation of large sets of genes [11]. Typical study designs comprise either PBMC cultures stimulated with mitogens [12], [13] or the investigation of blood cells or immune tissue after pathogen infection [14]–[16] or vaccination [17].

Concerning the porcine immune response on the transcriptomic level, our study was addressed to piglets after weaning which often suffer from production diseases caused by various factors including multiple facultative pathogens. To get a genome-wide comprehensive insight into *in vivo* transcriptional changes during the immune response, weaned piglets were vaccinated using tetanus toxoid (TT) as a model antigen for immune stimulation. TT vaccines are known to trigger both the cellular (Th1) and the humoral branch (Th2) of the immune system [18], [19] and represent a non-ubiquitous antigen for which weaning piglets are considered as antigen-naïve [17]. Thus, to identify gene transcripts with different abundances and their networks that are altered in leukocytes during the *in vivo* response to a model vaccine inducing a mixed Th1/Th2 response expression profiles of PBMCs before and several time points after immunization are displayed via genome-wide microarrays.

## Materials and Methods

### Animals, Vaccination and Blood Sampling

Animals used were owned by the Leibniz-Institute for Farm Animal Biology which gave the permission to perform the study. Animal care and tissue collection procedures followed the guidelines of the German Law of Animal Protection and the experimental protocol was approved by the Animal Care Committee of the Leibniz Institute of Farm Animal Biology and the State Mecklenburg-Vorpommern (Landesamt für Landwirtschaft, Lebensmittelsicherheit und Fischerei; LALLF M-V/TSD/7221.3-2.1-020/09).

**Figure 1:**
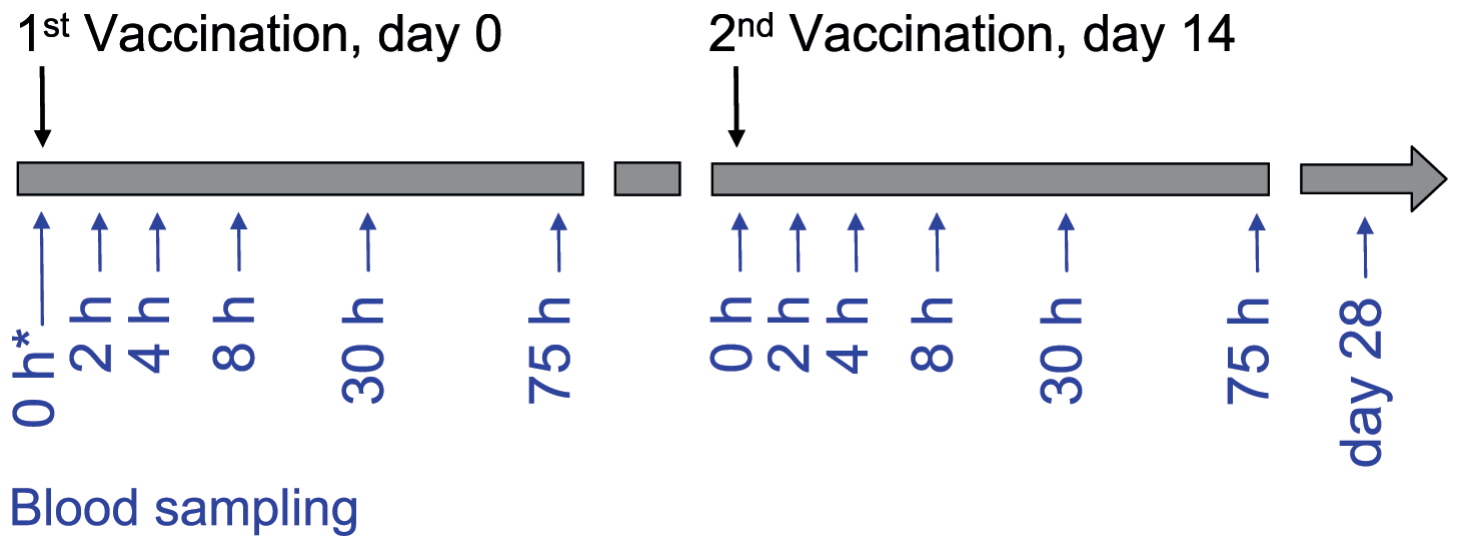
Experimental Design. Animals were vaccinated twice with TT. Directly before (0), as well as 2, 4, 8, 24 and 75 hours after each vaccination blood samples were collected. A final blood sample was taken at day 28. Pairwise comparisons of the reference time 0 hours against each following sampling time point were set up for the identification of DE-genes. * reference time point.

One week after weaning at an age of five weeks three male and three female piglets from three respective litters (n = 18) of a German Landrace herd were vaccinated subcutaneously with 1 ml (30 IU) of tetanus vaccine, composed of TT and aluminium hydroxide as adjuvant (Equilis Tetanus-Vaccine, Intervet, Unterschleißheim, Germany). Directly before (0), as well as 2, 4, 8, 24 and 75 hours after vaccination blood samples were collected (Figure 1). Vaccination and blood sampling were replicated 14 days after the first vaccination, a final blood sample was taken at day 28 when animals of our population reached antibody titers of 0.33 IU/ml with a standard deviation of 0.23 IU/ml.

### RNA isolation, Target Preparation and Microarray Hybridization

From 4 ml of blood PBMCs were isolated by centrifugation on a Histopaque (Sigma-Aldrich, Taufkirchen, Germany) density gradient. From the PBMCs preparations total RNA was isolated using TRI reagent (Sigma-Aldrich, Taufkirchen, Germany) followed by DNase treatment and a column based purification using the RNeasy Mini Kit (Qiagen, Hilden, Germany). RNA integrity was checked by visualization on 1% agarose gel containing ethidium bromide and the concentration was quantified by a NanoDrop ND-1000 spectrometer (PEQLAB, Erlangen, Germany). The absence of DNA contamination was tested by a PCR amplification of the porcine GAPDH gene (forward primer 5′-AAGCAGGGATGATGTTCTGG-3′; reverse primer 5′-ATGCCTCCTGTACCACCAAC-3′). All RNA was stored at -80°C until downstream preparation was performed. For each sampling time point three RNA pools were generated each of six individuals, one male and one female from the three respective litters. Biotin-labeled cRNA was synthesized using the GeneChip 3′ IVT Express Kit (Affymetrix, Santa Clara, CA, USA). Fragmented cRNA was hybridized on Affymetrix GeneChip Porcine Genome Arrays. After staining and washing the arrays were scanned and raw data were obtained using Affymetrix GCOS 1.1.1 software. According to the MIAME standard the microarray data has been deposited in the database of the National Center for Biotechnology Information Gene Expression Omnibus http://www.ncbi.nlm.nih.gov/geo [GEO: GSE38602].

### Data and Pathway Analysis

First, the microarray raw data were quality controlled by the MAS5 and normalized by the PLIER algorithm using Affymetrix Expression Console 1.1 software (Affymetrix, St. Clara, CA, USA). Pairwise comparisons of the three pools of the reference time of 0 hours against the respective following time points after the first and second vaccination were set up (Figure 1). Each comparison was subjected to a variance filter (TM4 Microarray Software Suite, [20], [21]) leaving 9000 probe sets of high variance. Afterwards, for each probe set a paired t-test of each comparison was performed. Resulting p values of each comparison were converted to q values, a false discovery rate estimation proposed by Storey and Tibshirani [22]. Bioinformatic analysis of significantly regulated transcripts (p<0.05) was carried out by use of a recent annotation of Affymetrix probe sets [23] and the Ingenuity Pathways Analysis (IPA) Software [24].

### Quantitative Real-time PCR

For validation of microarray data, the gene expression of five selected genes was determined using the same sample pools of cDNA used for microarray analyses. These cDNA pools were amplified by quantitative real-time PCR on an iQ5 PCR system (Bio-Rad Laboratories, München, Germany). Each amplification was done in duplicate in a final volume of 20 µl with 10 µl of LightCycler 480 SYBR Green I Master (Roche, Mannheim, Germany), 7.7 µl of Aqua dest., 0.4 µl of each primer (10 µM) and 30 ng (1.5 µl) cDNA. The amplified genes were KRAS, RPS6KB1, CD8A, CALR, STAT1, HPRT1 and PPIA (Table 1), while the last two were used as reference genes to account for variation of cDNA amounts after reverse transcription by calculating a normalization factor. Except for KRAS and HPTR1 the genes were amplified by nested PCR. A cDNA standard was amplified with the outer primer pair. The standard curve was derived from amplification of serial dilutions of the standard.

**Table 1:** Primer sequences used for quantitative real-time PCR.

Gene	Probe set ID	Outer primer sequence 5′-3′	Inner primer sequence 5′-3′
KRAS	Ssc.29092.1.A1_at	–	For TTCGTGTTCCCTCAATGTTTC Rev TGGTGCATGCAGTCAATTACT
RPS6KB1	Ssc.22127.1.S1_at	For TGATGAATGTCTTCCACAGTGA Rev GGAGAACATAGCAAGCAGCA	For CCTGCCTTAAAGAGCATTTCC Rev CGCACACTCAGACTGAAGACA
CD8A	Ssc.23489.1.S1_at	For CTGAATCCTGGAAAGTGAACAA Rev TCGGTCATAATTCTGTGTTTACAA	For CACGACCTCTAAAGGAAATCCA Rev CGAGGAGCACGTTCAAATATC
CALR	Ssc.3106.1.S1_at	For GGAGTTTGGCAACGAGACAT Rev AGGAATCTGGGGAGAGGAGA	For AGGCCAAGGATGAGCTGTAG Rev ACCAAATCCATCCCAAATCA
STAT1	Ssc.6025.2.A1_at	For CGGGGCATAAAAGTTGTGTT Rev CGGTTTCTCCTCAGTTTTGAA	For GGCTTTATGCTGCTGGCTAC Rev CTGGCTCCCTTGATAGAACTG
HPRT1*	Ssc.4158.1.S1_at	–	For GTGATAGATCCATTCCTATGACTGTAGA Rev TGAGAGATCATCTCCACCAATTACTT
PPIA*	Ssc.8046.1.A1_at	For AGCACTGGGGAGAAAGGATT Rev TGTCCACAGTCAGCAATGGT	For GATTTATGTGCCAGGGTGGT Rev CTTGGCAGTGCAAATGAAAA

* reference genes used for normalization.

## Results

We performed a microarray study of 12 sampling time points over a period of four weeks to get a comprehensive overview of gene expression changes during immune stimulation with TT as a model antigen. The examination of the transcriptomic response was performed with Affymetrix GeneChip Porcine Genome Arrays containing 24,123 probe sets of which 20,689 recently had been assigned to known porcine genes [23]. To analyse transcriptional changes pairwise comparisons were set-up of each time point after vaccination against 0 hours serving as the reference. Subsequently, by use of the IPA database for each time point after immunization canonical pathways were identified, which represented a significant number of genes with different transcript abundances (for simplicity, hereinafter referred to DE-genes).

### DE-genes after Tetanus Vaccination and Assignment to Canonical Pathways

To assign probesets to genes the microarray data of each comparison were submitted to the manually curated database Ingenuity Pathway Analysis [24]. The transcriptome data in total comprised more than 5000 DE-genes (p<0.05). Within the single time stages the numbers of DE-genes were between several hundred and more than 1000 with different proportions of up and down regulation (Table 2). IPA canonical pathways were identified for DE-genes (p<0.05, fold change >1.3) of each pairwise comparison of 0 hours to the respective time points after initial and second vaccination.

**Table 2:** Number, direction and q-values [34] of DE-genes (p<0.05) over the time points of blood sampling after vaccination.

Time after 1st vaccination	2 h	4 h	8 h	24 h	75 h	d 14
Number of DE-genes	679	1196	1104	773	549	642
increased transcript abundance	417	1063	889	233	118	207
decreased transcript abundance	262	133	215	540	431	435
q-value (at p = 0.05)	0.24	0.08	0.08	0.22	0.36	0.21
**Time after 2nd vaccination**	**2 h**	**4 h**	**8 h**	**24 h**	**75 h**	**d 28**
Number of DE-genes	485	358	824	942	1121	527
increased transcript abundance	301	187	406	557	259	408
decreased transcript abundance	184	171	418	385	862	119
q-value (at p = 0.05)	0.49	0.59	0.24	0.15	0.09	0.32

Here, we examined the transcriptional response at four time points within one day after each vaccination and the later response at 75 h and day 14. Concerning the former, i.e. the early response from two to 24 hours after vaccination, a top 20 list of canonical pathways was set-up derived by rank sums over the respective four time points (Tables 3 and 4). Based on individual rankings of pathways within the respective time points the rank sum represents the top pathways, which were present either repeatedly during day one or predominantly at single time points.

**Table 3:** Top 20 canonical pathways within the first 24 h of immune response after the first vaccination.

Ingenuity Canonical Pathway	Pathway Category	−log p at 2 h	−log p at 4 h	−log p at 8 h	−log p at 24 h
Fcγ Receptor-mediated Phagocytosis inMacrophages and Monocytes	1	6.79	2.25	2.37	6
CD28 Signaling in T Helper Cells	1	4.92	n.s.	1.68	5.63
CTLA4 Signaling in Cytotoxic T Lymphocytes	1	3.64	n.s.	n.s.	6.06
T Cell Receptor Signaling	1	2.2	n.s.	3.22	5.79
Regulation of IL-2 Expression in Activated andAnergic T Lymphocytes	1; 2	1.48	n.s.	4.13	2.31
Clathrin-mediated Endocytosis Signaling	1; 3; 9	4.15	2.66	n.s.	4.63
ILK Signaling	4	4.69	2.03	n.s.	1.59
FAK Signaling	4	3.83	1.58	1.68	4
VEGF Signaling	4; 10	3.11	2.98	2.6	4.74
IGF-1 Signaling	4; 10	1.61	3.5	2.63	3.54
Integrin Signaling	4; 5; 7	6.2	2.74	2.94	4.08
Protein Ubiquitination Pathway	5	1.45	3.7	5.69	n.s.
ERK/MAPK Signaling	5	3.01	2.05	4.26	5.09
Glucocorticoid Receptor Signaling	5	2.59	2.95	4.76	3.65
Estrogen Receptor Signaling	6	n.s.	3.82	4.02	n.s.
Aldosterone Signaling in Epithelial Cells	6; 12	2.6	2.47	3.06	2.12
NRF2-mediated Oxidative Stress Response	8; 13	2.11	2.14	5.93	1.66
Actin Cytoskeleton Signaling	9	5.15	3.11	4.62	4.81
Regulation of Actin-based Motility by Rho	11	3.94	3.68	1.36	1.62
Ephrin Receptor Signaling	9	2.71	n.s.	n.s.	5.45

1 Cellular Immune Response.

2 Cytokine Signaling.

3 Pathogen-Influenced Signaling.

4 Cellular Growth, Proliferation and Development.

5 Intracellular and Second Messenger Signaling.

6 Nuclear Receptor Signaling.

7 Cell Cycle Regulation.

8 Cellular Stress and Injury.

9 Organismal Growth and Development.

10 Growth Factor Signaling.

11 Neurotransmitters and Other Nervous System Signaling.

12 Cardiovascular Signaling.

13 Ingenuity Toxicity List Pathways.

n.s. not significant.

**Table 4:** Top 20 canonical pathways within 24 h of immune response after the second vaccination on day 14.

Ingenuity Canonical Pathway	Pathway Category	−log p at 2 h	−log p at 4 h	−log p at 8 h	−log p at 24 h
Fcγ Receptor-mediated Phagocytosis inMacrophages and Monocytes	1	n.s.	n.s.	3.77	3.36
fMLP Signaling in Neutrophils	1; 3	n.s.	n.s.	3.8	2.82
Clathrin-mediated Endocytosis Signaling	1; 4; 10	1.69	n.s.	4.88	2.78
Tumoricidal Function of Hepatic Natural Killer Cells	1; 6	1.64	1.54	n.s.	n.s.
B Cell Receptor Signaling	2	n.s.	n.s.	3.18	3.23
Role of MAPK Signaling in the Pathogenesis of Influenza	4; 5	1.62	1.48	1.84	1.63
Atherosclerosis Signaling	5; 13	n.s.	2.39	n.s.	n.s.
SAPK/JNK Signaling	6	n.s.	n.s.	5.84	2.75
Integrin Signaling	7; 8; 9	2.46	2.15	3.69	3.59
ILK Signaling	7	n.s.	n.s.	5.17	1.84
VEGF Signaling	7; 11	n.s.	n.s.	4.04	4.27
Protein Kinase A Signaling	8	n.s.	3.74	4.31	n.s.
Glucocorticoid Receptor Signaling	8	n.s.	n.s.	2.37	3.79
Insulin Receptor Signaling	8	2.05	n.s.	1.65	1.77
Actin Cytoskeleton Signaling	10	2.3	n.s.	5.37	5.16
Ephrin Receptor Signaling	10	2.15	1.35	1.66	n.s.
Regulation of Actin-based Motility by Rho	12	1.31	n.s.	2.87	3.13
Inhibition of Angiogenesis by TSP1	13	2.36	n.s.	3.21	n.s.
Aminosugars Metabolism	14	n.s.	2.9	n.s.	n.s.
N-Glycan Degradation	15	n.s.	2.48	n.s.	n.s.

1 Cellular Immune Response.

2 Humoral Immune Response.

3 Cytokine Signaling.

4 Pathogen-Influenced Signaling.

5 Disease-Specific Pathways.

6 Apoptosis.

7 Cellular Growth, Proliferation and Development.

8 Intracellular and Second Messenger Signaling.

9 Cell Cycle Regulation.

10 Organismal Growth and Development.

11 Growth Factor Signaling.

12 Neurotransmitters and Other Nervous System Signaling.

13 Cardiovascular Signaling.

14 Carbohydrate Metabolism.

15 Glycan Biosynthesis and Metabolism.

n.s. not significant.

### First Vaccination

Significant changes in the trancript abundances of PBMCs already occurred very early after *in vivo* immune stimulation. At 2 h after vaccination 679 DE-genes corresponding to 110 canonical pathways of immune response and other multiple cellular functions were found (see Dataset S1).

A considerable high number of transcripts with different abundance has been found at 4 h (1196 DE-genes) and 8 h (1104 DE-genes), that could be assigned to 72 and 99 canonical pathways, respectively. At 24 h after the first immunization 773 DE-genes were found related to 148 canonical pathways.

**Figure 2:**
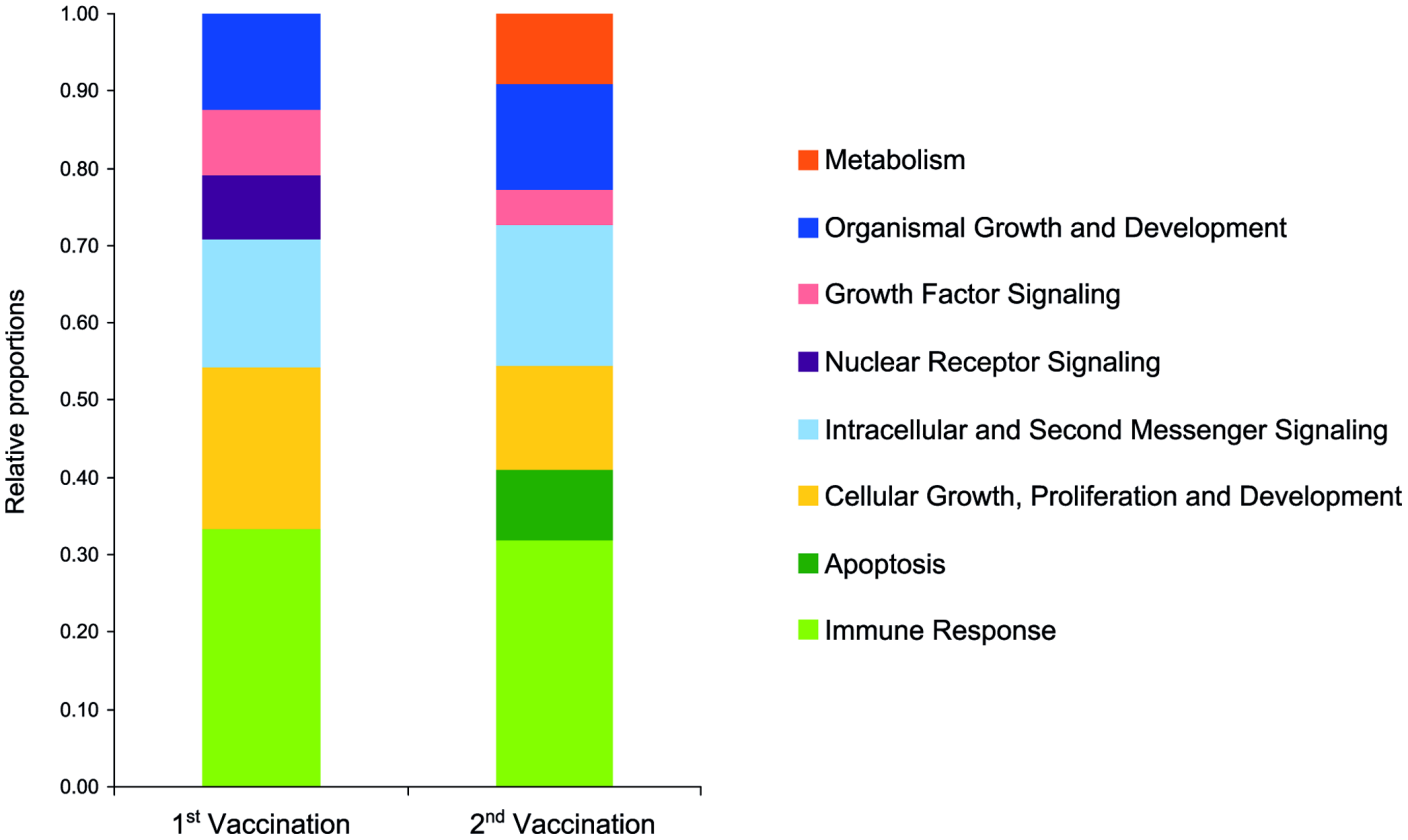
Most affected biofunctions within 24 hours after the first and after the second vaccination. Segments of the respective bars for the first and the second vaccination represent the relative frequencies of IPA biofunctional categories superior to canonical pathways that are most significant at 2 h, 4 h, 8 h and 24 h after immunization.

Concerning these four time points, i.e. within one day after vaccination, signaling pathways of multiple biological function were present (Table 3). Pathways of ‘cellular immune response’, ‘cellular growth, proliferation and development’ and ‘intracellular and second messenger signaling’ as well as ‘organismal growth and development’ were predominant (Figure 2).

75 h after the first vaccination for 118 increasingly- and 431 decreasingly abundant DE-genes a number of 37 canonical pathways were found. Pathways of the categories ‘cellular’ and ‘organismal growth and development’ as well as ‘intracellular and second messenger signaling’ were predominant.

At 14 days after the first vaccination, i.e. directly before the second vaccination, 207 increasingly abundant and 435 decreasingly abundant DE-genes were present. These genes correspond to 53 canonical pathways mostly represented by ‘cellular immune response’ or ‘cytokine signaling’ processes and ‘intracellular and second messenger signaling’ as well.

### Second Vaccination

2 h after the second vaccination 485 DE-genes were observed related to 19 canonical pathways, at 4 h 358 DE-genes correspond to 27 affected canonical pathways. The number of DE-genes gained to 824, 942 and 1121 at 8 h, 24 h and 75 h respectively. These genes were found associated with 155, 97 and 179 canonical pathways respectively, mostly represented by ‘cellular immune response’, ‘cellular’ and ‘organismal growth and development’ as well as ‘intracellular and second messenger signaling’ (Table 4, Figure 2).

For the final sampling 14 days after the second vaccination the numbers of DE-genes were 408 for increasingly abundant and 119 for decreasingly abundant related to 39 canonical pathways with predominant functions in immune response and ‘cellular growth, proliferation and development’.

Certain pathways were found to be affected over nearly all time points including integrin, VEGF, actin cytoskeleton and glucocorticoid receptor signaling. The transcriptional responses at four time points at day one after each vaccination are summarized in Tables 3 and 4. A comparison of the most frequently observed categories of these canonical pathways is illustrated by Figure 2. For both vaccinations canonical pathways of immune response were predominant followed by ‘cellular growth, proliferation and development’ and ‘intracellular and second messenger signaling’. For the second vaccination among the top ranking also pathways of apoptosis and metabolism are present.

### Validation by Quantitative Real-time PCR

For five selected genes the expression values of the microarray data were validated by comparison to copy numbers determined by quantitative real-time PCR. Relative transcript abundance of three genes were significantly correlated at 0.42 to 0.58; for two genes a moderate correlation at 0.35 to 0.36 was observed (Table 5).

**Table 5:** Correlation between microarray gene expression and results of quantitative real-time PCR for selected genes.

Gene	Spearman’s rho	p-value	Number of involved canonical pathways
KRAS	0.58	<0.01	92
RPS6KB1	0.58	<0.01	20
CD8A	0.35	0.07	3
CALR	0.36	0.06	4
STAT1	0.42	0.04	26

## Discussion

The objective of this study was to evaluate global transcriptional *in vivo* response of porcine PBMCs to immune stimulation using TT as a T cell dependent model antigen triggering a mixed Th1/Th2 immune reaction. Our results revealed a large number of genes with differential transcript abundance. The bioinformatic analysis of these genes via the ingenuity knowledge base displayed differential levels of transcripts assigned to numerous canonical pathways. Among these, with regard to immune function, ‘cellular immune response’ and ‘cytokine signaling’ pathways were predominant. In terms of further functions the activity of PBMCs was mainly made up of ‘cellular growth, proliferation and development’, ‘intracellular and second messenger signaling’ as well as ‘organismal growth and development’.

Our microarray data show that *in vivo* already very early after TT vaccination, i.e. at 2 h, 4 h and 8 h, considerable changes on the mRNA level occurred. Using a cDNA expression array with 588 genes Regnström and colleagues observed an immediate transcriptional response of murine spleen cells already 4 h after *in vitro* restimulation by TT covering Th1 and Th2 markers. [25]. *In vivo*, we observed an even earlier broad transcriptional response of naïve PBMC. Among the Top 20 canonical pathways at day one after the initial vaccination (Table 3) the signaling pathway ‘regulation of IL-2 expression in activated and anergic T lymphocytes’ indicates the early occurence of cytokine signaling events. In addition, IL-2 signaling and other cytokine pathways, IL-3, IL-4 and IFNγ signaling were found among the predominant pathways following each vaccination. The T cell growth factor IL-2 is known to induce the proliferation of T cells autocrinally as well as the proliferation of B cells. Produced by activated T cells it is the most important cytokine for the development of adaptive immune responses. IL-2 pathways featured almost up shifted transcripts within day one after the initial vaccination. At 8 h after the first vaccination as key molecules of these pathways we found increasead transcript abundance for the CD3 receptor and the ELK1 transcription factor for IL-2 expression (see Dataset S1).

Likewise, several T cell costimulatory pathways in particular ‘CD28 signaling in T helper cells’, ‘CTLA4 signaling in cytotoxic T lymphocytes’ and ‘T cell receptor (TCR) signaling’ showed a considerable response early at 2 h, 8 h and 24 h (Table 3) as well as moderate responding after the second vaccination (see Dataset S1). T cell receptor (TCR) signaling is responsible for signal transduction after MHC associated antigenes are recognized and bound by the TCR. Following the first vaccination we found transcripts of the costimulatory receptors CD4 increased and CD28 decreased (both at 2 h) and for the CD3 receptor increased (at 8 h) which is part of the TCR-CD3 complex. The binding of antigens to TCR leads to a sequence of tyrosinase activity eventually resulting in transcriptional activation of several genes. For one of these downstream signaling processes we found increased transcript abundance of NFAT, a transcription factor of the IL-2 gene at 24 h after the second vaccination.

The costimulatory pathways ‘CD28 signaling in T helper cells’ and ‘CTLA4 signaling in cytotoxic T lymphocytes’ generally represent antagonistic processes in T cells. CD28 acts as a positive costimulatory receptor for B7 molecules on antigen presenting cells whereas CTLA4, which is also a receptor for B7, is known to alter costimulatory to inhibitory signals. However, we did not find changes of transcript quantities of CTLA4 and associated negative-signaling proteins at the early time points rather decreased transcript abundance after the second vaccination of SHP1 and CTLA4 at 8 h and 24 h, respectively.

For CD28 Signaling we hardly found a conclusive alteration of pathway components at early time points, except for transcript increase of calmodulin, calcineurin and NFAT (activated by calmodulin-calcineurin interaction) indicating activated IL-2 transcription at 24 h after second vaccination. In addition, we observed an obvious down regulation at day three (75 h - second vaccination) with ten molecules of decreased transcript abundance.

In our results based on Ingenuity Pathway Analysis the response of PBMCs was mainly made up of cellular immune response and cytokine signaling. Signaling pathways of the humoral immune response were not found among the predominant. However, it should be mentioned here that the ingenuity category for humoral immune response listed only 17, whereas the category cellular immune response listed 70 canonical pathways with some overlap between the two groups. Consequently, the category humoral immune response may appear under-represented, although a number of DE-genes involved in humoral immune events were found. For instance the humoral immune response pathway ‘B Cell Receptor Signaling’ was found regulated at six time points.

TT vaccines are known to trigger a mixed Th1 and Th2 immune response [18], [19] and represent a non-ubiquitous antigen which has been used as model antigen here. To stimulate an *in vivo* immune response in pig, tetanus vaccine was administered to piglets. In general, in addition to the toxoid, vaccines contain aluminum adjuvants generally known as immunostimulators for the Th2 type of immune response [26]. For tetanus vaccination it has been shown that it is only the combination of TT and adjuvant, which causes an effective immune response [25], [27], [28].

Consistent with our findings are earlier results for the pig [17], [29] and studies of murine spleen lumphocyctes [25], [28] that found differential expression of genes involved in both immune response and processes of cell signaling, cellular and organismal growth, cell cycle control, apoptosis, cytoskeleton organization, biosynthesis, metabolism as well as stress, toxicology response and oncogenesis or tumor suppression.

Moreover, our microarray results and the aforesaid studies in mice demonstrate clearly an early onset of transcriptional responses to immune stimulation within few hours that had also been shown for murine T cell stimulation with superantigen [30]. However, we also found evidence that the early response after the second vaccination was less pronounced given the fact that only few and hardly any immune specific canonical pathways could be found at 2 h after the booster vaccination. Further, we observed indications that the response to the second vaccination was prolonged in terms of the duration of shifts of transcript abundance. In fact, pathways found at 75 h after the initial vaccination were hardly found among the pathways affected during the first 24 h. However, after the booster vaccination the time point 75 h shared multiple pathways with the previous samplings.

Disease resistance and immune competence are heritable [31], [32] but difficult to measure and hence to address by breeding. QTL mapping of cellular and humoral immune traits [33]–[35] in experimental cross breeds facilitates the identification of candidate genes for immune competence. Recently QTL for TT antibodies were detected on SSC 2, 4, 8, 11 and 18 [36]. Beside the mapping approach gene expression profiling of immune tissues in defined phenotypes enables the detection of functional networks and thus functional candidate genes. Concerning both approaches additional research is needed to further characterize these candidate genes to reveal genetic markers for selection of animals endogenously better protected against infection.

## Supporting Information

Dataset S1 **Significant IPA canonical pathways and respective DE-genes (p<0.05, FC >1.3).** Excel file with 12 spreadsheets for 12 time points after vaccination and one spreadsheet listing DE-genes over all time points.
(XLS)
